# Attitudes towards digital health technology for the care of people with chronic kidney disease: A technology acceptance model exploration

**DOI:** 10.1371/journal.pdig.0000614

**Published:** 2024-10-09

**Authors:** Daphne Kaklamanou, Le Nguyen, Miznah Al-Abbadey, Nick Sangala, Robert Lewis

**Affiliations:** 1 School of Psychology, Sport and Health Sciences, University of Portsmouth, Portsmouth, United Kingdom; 2 Chronic Pain Service, St Mary’s Hospital, Isle of Wight NHS Trust, Newport, United Kingdom; 3 Queen Alexandra Hospital, Portsmouth Hospital University Trust, Portsmouth, United Kingdom; Iran University of Medical Sciences, ISLAMIC REPUBLIC OF IRAN

## Abstract

**Background:**

Chronic Kidney Disease (CKD) is a long-term condition and a major health problem, which affects over 3.5 million adults in the UK. Use of digital technology has been proposed as a means of improving patient management. It is important to understand the factors that affect the acceptability of this technology to people living with chronic kidney disease. This study used the Technology Acceptance Model 3 (TAM) to investigate whether perceived ease of use and perceived usefulness could predict intention behaviour. It then investigated if intention to use digital technology predicted actual use.

**Methodology:**

This was a cross-sectional study whereby the TAM3 questionnaire was sent online to people known to have chronic kidney disease via Kidney Care UK. The characteristics of the respondents (age, sex, CKD stage) were recorded.

**Principal Findings:**

The questionnaire was sent to 12,399 people, of which 229 (39% drop out) completed it. The respondents’ age ranged from 24–90 years and 45% (n = 102) were male. Thirty-five percent of participants had advanced kidney care, 33% (n = 76) had kidney transplant and 22% (n = 51) had CKD. A multiple regression analysis showed a perceived ease of use and perceived usefulness of the technology predicted behaviour intention to use digital health technology. Behaviour intention did not significantly predict actual use behaviour.

**Conclusion:**

Perceived usefulness and perceived ease of use are important factors in determining the intention of people with CKD to use digital healthcare. However, a gap exists between this intention and readiness to actually use the technology. This needs to be overcome if digital healthcare is to gain future traction in the clinical scenario.

## Author summary

A previously validated online questionnaire (Technology Acceptance Model 3) was used to explore how people with Chronic Kidney Disease (CKD) perceive digital health technology and to assess their attitudes towards its use in the management of their condition. All participants had CKD at various stages of severity or were recipients of a kidney transplant and were receiving specialist care at the Wessex Kidney Centre based in Portsmouth UK. Two-hundred and twenty-nine people aged 24–90 completed the survey. Responses were unaffected by the type of kidney disease or the sex of the respondent. We found that people perceived digital technology to be easier to use if organisational and technical resources were in place to support its use, if they were less anxious and if they enjoyed using it. Participants who reported digital health technology easy were those who also considered it to be highly relevant to their care. Intention to use digital health technology was greater when participants perceived it to be easy to use and useful. This effect was amplified by age, with younger people being more likely to use digital health technology than older people.

## Introduction

Chronic Kidney Disease (CKD) in known to affect over 3.5 million people in the UK [1] although many cases of CKD go undiagnosed [2]. CKD can have a significant impact on a person’s quality of life and can lead to life-changing treatments such as renal replacement therapy. Management of CKD also places a financial burden on individuals and communities. The National Health Service (NHS) spent £6.4 billion on kidney care in 2023, 3.2% of the NHS budget [3]. In addition to healthcare costs, people with CKD and their carers may suffer financial loss from missing working days [4].

CKD is defined as the presence of impaired kidney function or protein in the urine for 3 months or more [1,5]. The condition is usually progressive and is therefore classified into five stages of severity [6] based on the estimated glomerular filtration rate (eGFR).

There are two major impacts of CKD on an individual; it increases the risk of cardiovascular disease (in the majority) and may progress to kidney failure (in the minority). To reduce this risk, people with CKD need to adopt a healthy lifestyle (healthy diet, weight control physical exercise etc.; [7]. Risk factors for progression also need to be identified and managed at an early stage. The most important of these is the need for tight blood pressure control, which requires regular monitoring. Many of these interventions can be managed by patients themselves if they are given the correct guidance and adequate support by healthcare professionals. By giving more control of management to people with CKD, there is a potential to improve outcomes [8] whilst reducing unnecessary use of healthcare resources. Specialist input into management of people with CKD can thereby be reduced.

Digital health technology, such as apps, wearable devices, mobile health, telehealth, and telemedicine [9] provides a means of improving expert support for people living with CKD, as well as improving access to health care services and integrating communication [10]. In the last twenty years the use of smartphones has grown such that 66% (5.22 billion) of the world’s population use them on a regular basis [11]. These may provide a means of communication for various digital health technologies which can utilise electronically captured data, cloud computing, artificial intelligence, machine learning, digitally mediated diagnostics and treatment, telehealth, and consumer-facing mobile health applications to name a few [12]. All these advances may allow earlier diagnoses and interventions, improved health outcomes and support for self-management [13]. Digital health technology facilitates a person-centred approach which allows people take a central role in the management and care of their condition [14]. Studies have already shown that digital health technologies can improve health outcomes and promote healthy behaviour. For instance, users of health apps are more likely to track their health-related goals and engage in health-promoting behaviours than non-health app users [15]. In a systematic review of the effectiveness of digital health records, the reported benefits included improvements in disease knowledge, patient engagement, treatment adherence, self-management and self-efficacy [16]. Despite these apparent benefits, and that the UK public is supportive of its many uses [17] digital platforms designed specifically for people with CKD have been slow to develop.

The ambition to encourage digital technologies for healthcare is one aim of the NHS Long Term Plan [18]. An app called Patients Knows Best is now in use to deliver this ambition in a generic way across a variety, disease areas (e.g. heart failure, prostate cancer, cystic fibrosis; [19]. Patient Knows Best (PBK) is a single platform than one can share their health information electronic records and allows access to their own data. PBK does not have an interface between patients, carers and health professionals. Another platform, the MyRenalCare web-app [20] is specifically designed for people with kidney disease and acts as an interface between patients and health professionals. It is possible that digital technologies designed for specific disease areas provides more focused care for patients, but this remains unexplored. In a systematic review identifying barriers and enablers to the diagnosis and management of CKD, one of the enablers was supportive technology (e.g. scanned records; [21] in the management of the condition [22]. So, it would seem that there is a potential role for technology in CKD management. There have been studies looking at acceptability of specific digital health technologies such as wearables [23] to support the self-management of CKD, but no study has explored the patients’ attitudes to the concept of digital health technology.

Exploring the factors influencing the acceptability of digital health technology, especially within a condition such as CKD where self-management and continuous monitoring is an important aspect of the care, is important if such technologies are to be adopted. This is the purpose of this study.

### Technology acceptance model

This study uses the Technology Acceptance Model (TAM3) [24] to explore factors that can influence people with CKD’s intention to use digital health technology in the management of their condition and is influenced by its perceived usefulness and ease of use. Perceived usefulness is defined as the extent to which a person believes that utilising technology can help improve their performance. Perceived ease of use is defined as the extent to which a person believes that technology can be used effortlessly. A systematic review of 32 studies found that the TAM and the modified TAM models are still valid in predicting acceptance and use of remote care technologies by healthcare professionals [25], as well as within different types of health professionals.

Perceived ease of use is characterised by five factors [24]. Perceptions of external control suggests the degree to which an individual believes that a digital health technology has the right organisational and technical resources to support its use. Previous research on digital health technology identified the lack of organisational or technical resources for an app as part of the competency frustration people may have in using the technology, such as the work syncing among devices [26]. The next factor is computer anxiety and that is the fear one might experience when using the digital health technology. Fear of using digital health technology has been found to be one of the patient level barriers to the uptake of digital care in cardiovascular disease [27]. Computer playfulness is the degree to which people are able to interact with the digital health technology in a less cognitive demanding way. People with stroke have emphasised the importance of the digital health technology having an interface that is easy to use such as larger screens [28]. Computer self-efficacy, which is the degree to one believing that they can perform a task using the digital health technology. In a study with patients from disadvantaged background, self-efficacy and perceived ease of use were the significant themes in terms of whether they would be using the remote monitoring of chronic diseases [29]. The final factor that contributes to perceived ease of use is the perceived enjoyment. This applies to the enjoyment people experience from the digital health technology irrelevant to the effects it might have towards the management of the condition (e.g. gamification or the online communities provided by the digital health technology). All of the factors focus on the use of the digital health technology, and it’s use. According to the TAM, the next factor that contributes to the continuous use of the digital health technology is its perceived usefulness.

Perceived usefulness is characterised from six factors. Perceived ease of use is the first factor and is defined as the degree to which one believes using the digital health technology will be free of effort. Subjective norm is the second factor that looks at whether the people that are important to one (e.g. family, friends, doctors, healthcare professionals) will think it is important for them to use the digital health technology. When looking at an electronic coach targeting self-management in people with type 2 diabetes, subjective norm was one of the predictors in why people accepted the technology [30]. Image is the degree to which an individual believes that using the digital health technology will improve their social status. The next factor is job relevance, and it states to how applicable one finds the digital health technology to their health care. Output quality refers to the degree that one believes that the digital health technology is performing their tasks well. Results demonstrability is the degree to which one believes that the results of using the digital health technology is observable, communicable. For example, people are observing a difference to the management of their condition through the digital health technology.

The current study is using an established online questionnaire, based on the TAM3, to identify factors that impact acceptability and intention to use digital health technology to manage health in people with CKD. This is to inform the implementation of digital technology in CKD care in the future. By understanding these factors, digital health technology can be developed to meet the needs of people with CKD. Ultimately, this research aims to inform a more efficient and cost-saving solution for managing CKD.

## Methods

### Participants

The UKs major charity for people living with CKD, Kidney Care UK, provided their support for the process of recruiting participants. They distributed a link to the questionnaire to 12,399 people with kidney disease and were able to tell when the link was accessed. People were informed that to participate in the study, they needed to meet the following criteria: aged 18 or over, currently have CKD and can provide informed consent. The target number of participants needed to obtain sufficient data for significant findings was 216. This target was established with a power calculation using the G*Power application. Analysis of access to the link showed that a total of 587 people started the survey and 229 people completed it (39% drop out). The high drop-out rate was attributed to participants accessing the study out of interest in the prize draw but then not continuing with the questionnaire.

The participants’ age ranged from 24–90 years old (*M* = 61.24, *SD* = 12.83). A total of 53.60% (*n* = 118) were female and 46.40% (*n* = 102) were male, with no specified response in nine participants. The majority of the participants were White (English, Welsh, Scottish, British), 94.7% (*n* = 214), with 2.20% (*n* = 5) of Black ethnicity, 1.7% (*n* = 4) of South Asian and 0.9% (*n* = 2) of mixed heritage.

### Design and ethics

This was a cross-sectional study using an online questionnaire via Qualtrics. The study was approved by the Science & Health Faculty Ethics Committee by the University of Portsmouth (SHFEC App 2022–099).

### Materials

In this study, the questions were split into three themes to gather information from participants and were asked the question in the following order. The first theme asked about demographic information, like age and gender. The second theme asked about the participant’s diagnosis with chronic kidney disease. The third theme focused on asking questions based on the Technology Acceptance Model (TAM) 3 [24]. We used an established questionnaire with 51 items that explored whether people would be willing to use technology to help manage their condition. Previous research has established that it has good internal reliability with Cronbach’s alpha values ranging from α = 0.76–0.93 [31,32]. All items unless stated differently, were answered on a 7-point Likert scale totally disagree to totally agree. For examples, of the items please see the supplementary materials in the Open Science Framework site (https://osf.io/ntd86/?view_only=909135681907400284a46d94d876a0ff).

*Objective Usability*: Participants were asked to answer a single question on average, how much time do you spend on digital health technology a day? This was an open-ended question, asking them to indicate the hours and/or minutes.

*Behavioural Intention*: A total of three items were used to measure behavioural intention and the sum of those three items was calculated, indicating that a higher score people had a stronger intention. Cronbach’s alpha for the three items was excellent *α* = .96.

*Perceived Ease of Use*: A total of four items were used to measured perceived ease of use and the sum of those was calculated, indicating that the higher score the easier to use the digital health technology. The Cronbach’s alpha was excellent *α* = .96.

*Subjective Norm*: The sum of four items was used to calculate subjective norm. The Cronbach’s alpha was very good *α* = .81.

*Image*: The sum of three items was used to calculate the social status (image) of using digital health technology. The Cronbach’s alpha was excellent *α* = .91.

*Job Relevance*: The sum of three items was used to calculate job relevance. The Cronbach’s alpha was excellent *α* = .90.

*Output Quality*: The sum of three items was used to calculate output quality. The Cronbach’s alpha was very good *α* = .83.

*Results Demonstrability*: The sum of four items was used to calculate the score for results demonstrability. The Cronbach’s alpha was very good, *α* = .81.

*Computer Self-Efficacy*: A total of four items were used to calculate the sum, with a higher score the more self-efficacy one has. The Cronbach’s alpha was very good, *α* = .81.

*Perceptions of External Control*: The sum of four items was calculated to derive a score for perceptions of external control, with a higher score indicating a higher perception of external control. The Cronbach’s alpha was questionable *α* = .68.

*Computer Anxiety*: The sum of four items was used to measure anxiety about digital health technology. The Cronbach’s alpha was excellent *α* = .93.

*Computer Playfulness*: A total of four items were used to measure playfulness with technology and the sum was calculated. The Cronbach’s alpha was excellent, *α* = .93.

Perceived Enjoyment: A total of three items were used to calculate the sum for the perceived enjoyment of digital health technology. The Cronbach’s alpha was excellent *α* = .92.

*Voluntariness*: A total of three items were used to measure how voluntary using the digital health technology would be. The Cronbach’s alpha was questionable, α = .66.

*Perceived Usefulness*: was measured using a total of four items and the sum of those was calculated. The Cronbach’s alpha was excellent, *α* = .93.

### Procedure

Participants were invited to participate in the study by clicking on the link provided in the Kidney Care UK newsletter. Before beginning the study, participants had the opportunity to review the Participant Information Sheet, which provided additional details about the study. If participants had any questions or concerns, they could contact the researchers for clarification. Once participants had read and understood the Participant Information Sheet, they were asked to provide informed consent (online via a tick box) before proceeding to the questionnaires. The questionnaires took approximately 15 minutes to complete. After completing the questionnaires, participants were directed to a Debrief form located at the end of the study. As a token of appreciation for their participation, participants were given the option to enter a prize draw by clicking on another link provided at the end of the study, that took them to a different survey and they could add their details. The prize draw had three prizes: £75 for first place, £50 for second place, and £25 for third place.

### Analysis

The data were collected on Qualtrics and analysed using the SPSS version 28.0 software. Responses with over 80% missing data were omitted before the analysis. Demographic information was first analysed to gain insights into the sample’s characteristics. Regression analyses were conducted to explore whether perceived usefulness and perceived ease of use could predict one’s intention to use technology.

## Results

### Demographic and diagnosis data

There were no gender differences between people’s intention to use digital health technology or any of the variables from the Technology Acceptance Model 3 indicating that both genders responded similarly to the acceptance of the digital health technology. However, males had a significantly higher number of hours of digital health technology use than female participants (*U* = 4,029.50, *Z* = -2.25, *p* = .024). Please see Table 1 for full results.

**Table 1:** Gender Differences in the Technology Acceptance Variables.

	Gender		
Technology Acceptance Variables	Male	Female	*p*
Computer Self-Efficacy	20.27 (5.81)	20.53 (4.46)	.724
Perception of External Control	19.52 (5.29)	19.65 (3.74)	.837
Computer Playfulness	15.74 (4.24)	15.71 (3.53)	.959
Computer Anxiety	10.16 (6.04)	10.73 (5.81)	.479
Perceived Enjoyment	12.74 (4.36)	12.76 (3.97)	.965
Subjective Norm	19.33 (4.91)	18.96 (4.23)	.552
Voluntariness	16.04 (3.60)	15.46 (2.98)	.193
Image	8.56 (4.43)	8.32 (4.41)	.690
Job Relevance	14.69 (4.40)	15.07 (4.06)	.518
Output Quality	13.83 (4.07)	13.32 (3.59)	.324
Results Demonstrability	20.58 (5.07)	20.64 (4.65)	.920
Perceived Usefulness	21.49 (6.08)	21.75 (5.48)	.733
Perceived Ease of Use	20.71 (6.32)	20.75 (5.67)	.952
Behavioural Intention	16.50 (4.89)	16.48 (4.16)	.972
Technology Use^a^	.50 (1.00)	.08 (1.00)	.024**

Note.

^a^. Median (Interquartile Range Reported) and Mann-Whitney U

** *p* < .050

Majority of the participants reported (21.1%, *n* = 48) reported to have an income between £20,000–30,000 and also preferred not to report the income (21.1%, *n* = 48). A total of 18% (*n* = 41) reported to have an income between £10,000–20,000, 13.2% (*n* = 30) reported to have an income between £40,000–50,000, 11.4% (*n* = 26) reported to have an income between £30,000–40,000, 83% (*n* = 19) reported to have an income above £50,000 and 7% (*n* = 16) reported to have an income below £10,000. In terms of education, majority of the participants had a high school diploma (36.6%, *n* = 83), 21.1% (*n* = 48) had a bachelor’s degree, 17.6% (*n* = 40) had some high school, no diploma, 13.7% (*n* = 31) had a Master’s degree, 8.4% (*n* = 19) had an ‘other diploma’ and 2.6% (*n* = 6) had a PhD.

Thirty-five percent (*n* = 81) of participants had advanced kidney care, 33% (*n* = 76) had kidney transplant and 22% (*n =* 51) had CKD. Due to the question being self-reported it is challenging to know the exact stage they were at. The duration of their diagnosis ranged from 1 to 76 years (*M* = 20.74, *SD* = 16.16). For the means of the items and the correlations see Table 2.

**Table 2:** Means (Standard Deviation) and Correlations of the Variables (*n* = 220).

	*M (SD)*	*α*	1	2	3	4	5	6	7	8	9	10	11	12	13	14	15
1.Computer Self-Efficacy	20.31 (5.27)	.81	-	.75*	.49*	-.45*	.55*	.54*	.31*	.03	.57*	.59*	.60*	.63*	.71*	.61*	.13
2.Perception of External Control	19.54 (4.65)	.68	-	-	.53*	-.51*	.65*	.59*	.33*	.04	.66*	.69*	.66*	.74*	.78*	.73*	.11
3. Computer Playfulness	15.64 (4.03)	.64	-	-	-	-.24*	.53*	.36*	.17**	.22*	.34*	.43*	.40*	.38*	.43*	.41*	.001
4. Computer Anxiety	10.50 (5.97)	.92	-	-	-	-	-.59*	-.46*	-.24*	.24*	-.54*	-.58*	-.73*	-.54*	-.67*	-.67*	-.05
5. Perceived Enjoyment	12.73 (4.28)	.92	-	-	-	-	-	.60*	.20**	.11	.65*	.68*	.65*	.61*	.67*	.70*	.07
6. Subjective Norm	19.10 (4.75)	.81	-	-	-	-	-	-	.27*	.12	.63*	.65*	.57*	.57*	.54*	.61*	.06
7. Voluntariness	15.64 (3.47)	.66	-	-	-	-	-	-	-	-.22*	.22*	.19*	.36*	.27*	.31*	.31*	-.04
8. Image	8.49 (4.38)	.91	-	-	-	-	-	-	-	-	.08	.14**	-.09	.003	-.06	-.07	-.005
9. Job Relevance	14.87 (4.30)	.90	-	-	-	-		-	-	-	-	.73*	.67*	.76*	.64*	.77*	.09
10. Output Quality	13.55 (3.93)	.83	-	-	-	-	-	-	-	-	-	-	.71*	.66*	.66*	.68*	.07
11. Results Demonstrability	20.53 (4.96)	.81	-	-	-	-	-	-	-	-	-	-	-	.65*	.75*	.78*	.08
12. Perceived Usefulness	21.50 (5.98)	.93	-	-	-	-	-	-	-	-	-	-	-	-	.77*	.82*	.07
13. Perceived Ease of Use	20.60 (4.66)	.96	-	-	-	-	-	-	-	-	-	-	-	-	-	.74*	.10
14. Behavioural Intention	16.41 (4.66)	.96	-	-	-	-	-	-	-	-	-	-	-	-	-	-	.10
15. Technology Use	2.27 (11.43)	-	-	-	-	-	-	-	-	-	-	-	-	-	-	-	-

Notes. 1. Computer Self Efficacy; 2. Perception of External Control; 3. Computer Playfulness; 4. Computer Anxiety; 5. Perceived Enjoyment; 6. Subjective Norm; 7. Voluntariness; 8. Image; 9. Job Relevance; 10. Output Quality; 11. Results Demonstrability; 12. Perceived Usefulness; 13. Perceived Ease of Use; 14. Behavioural Intention; 15. Technology Use

*p < .001

**p < .05

### Perceived ease of use

Multiple regression analysis was used to assess the contribution of the determinants on perceived ease of use. Computer self-efficacy, perception of external control, computer anxiety and perceived enjoyment were found to account for a significant degree of perceived ease of use *R*^2^_*Adjuste*d_ = .73, *F* (5, 206) = 115.71, *p* < .001 (see Table 3). This indicates that people who strongly believe that there is organisational structure to support the use of digital health technology, and report less anxiety about computers, but have a higher control beliefs about computers and a high sense of enjoyment about digital health technology are more likely to perceive the technology as easier to use.

**Table 3:** Multiple Regression Results of Hypothesised Predictors of ‘Perceived Ease of Use’ of Digital Health Technology.

Variable	*B*	Standardised *B*	*t* value	*p* level	*VIF*
Computer Self-Efficacy	.19	.16	2.94	.004	2.35
Perception of External Control	.56	.42	6.91	< .001	2.92
Computer Anxiety	-.28	-.29	-6.01	< .001	1.79
Computer Playfulness	-.08	-.05	-1.15	.250	1.61
Perceived Enjoyment	.23	.18	3.35	.001	2.27

### Perceived usefulness

Multiple regression analysis was used to assess the contribution of the determinants on perceived usefulness. Image, job relevance and output quality were found to account for a significant degree of perceived usefulness *R*^2^_*Adjuste*d_ = .65, *F* (6, 190) = 62.59, *p* < .001 (see Table 4). This means that people are more likely to find digital health technology useful if they perceive the technology as easy to use, of relevance to their Chronic Kidney Disease Care and feel they will be stigmatised if they do not use the technology.

**Table 4:** Multiple Regression Results of Hypothesised Predictors of ‘Perceived Usefulness’ of Digital Health Technology.

Variable	*B*	Standardised *B*	*t* value	*p* level	*VIF*
Perceived Ease of Use	.10	.24	3.87	< .001	2.08
Subjective Norm	.01	.002	.03	.257	2.04
Image	-.16	-.13	-2.85	.005	1.13
Job Relevance	.61	.47	6.84	< .001	2.67
Output Quality	.14	.10	1.35	.971	3.16
Results Demonstrability	.14	.08	1.72	.087	2.72

### Perceived usefulness and perceived ease of use on behavioural intention

Multiple regression analysis showed that perceived usefulness and perceived ease of use accounted for a significant degree of behavioural intention *R*^2^_*Adjuste*d_ = .70, *F*(2, 219) = 259.50, *p* < .001 (see Table 5). This indicates individuals are more likely to have higher intentions to use the digital health technology if it is seen as useful and easy to use.

**Table 5:** Multiple Regression Results of ‘Perceived Usefulness’ and ‘Perceived Ease of Use’ as Hypothesised Predictors of ‘Behavioural Intention’.

Variable	*B*	Standardised *B*	*t* value	*p* level	*VIF*
Perceived Usefulness	.48	.61	10.53	< .001	2.50
Perceived Ease of Use	.21	.27	4.63	< .001	2.50

However, further regression analysis found that behavioural intention did not significantly predict one’s actual use behaviour (*R*^*2*^ = .01, *F* (1, 203) = 1.87, *p* = .173)

### Age and behavioural intention

**Fig 1:**
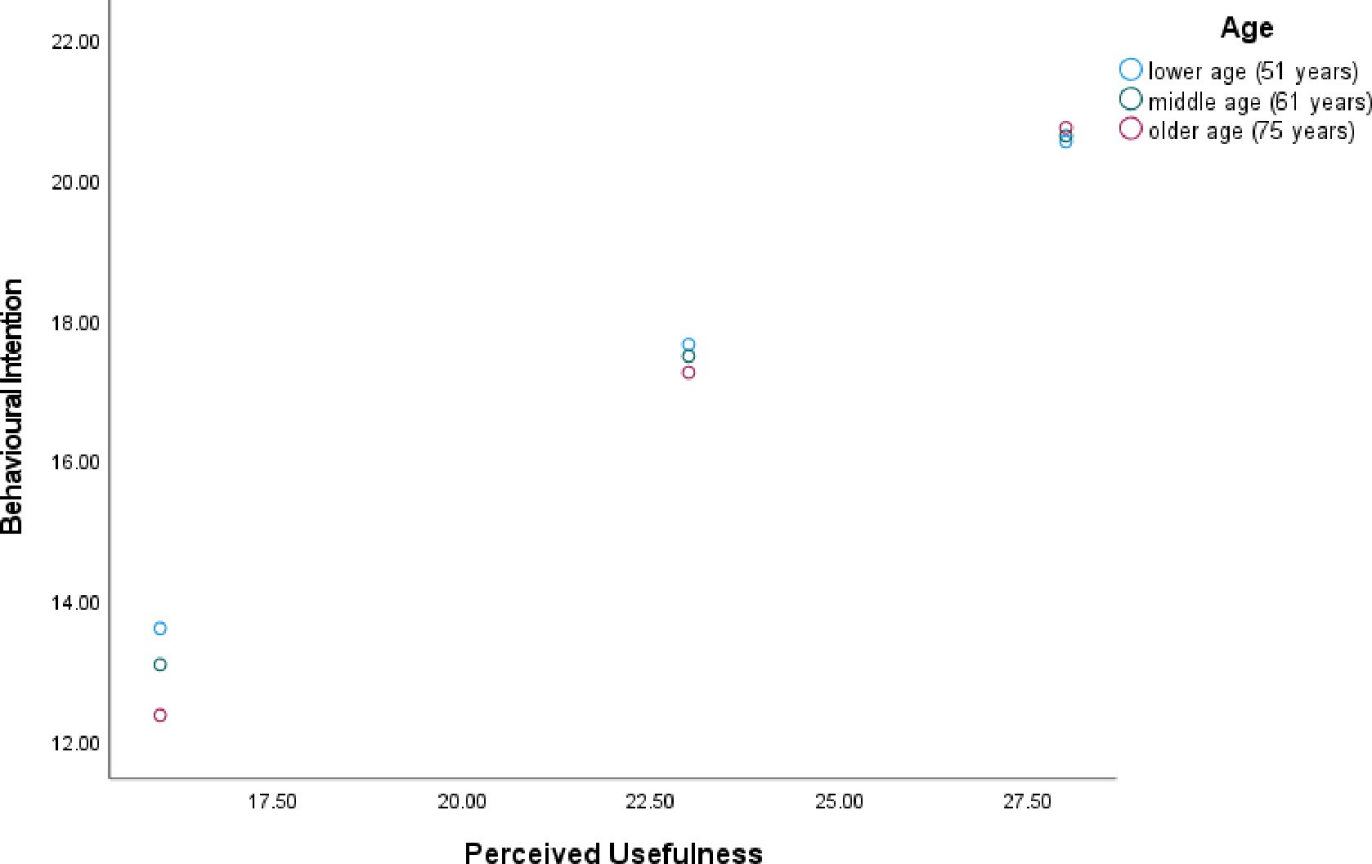
Interaction of Age and Perceived Usefulness on Behavioural Intention.

Previous studies have shown that age plays a role in predicting intention to use technology. Similarly, our analysis showed that age significantly predicted one’s intention to use digital health technology (*R*^*2*^_*Adjusted*_ = .05, *F*(1, 218) = 11.37, *p* < .001; see Table 6). This means that the younger the person is, the more likely they intend to use digital health technology for CKD. We investigated whether age moderated the relationship between perceived ease of use and behavioural intention and found that age was not a significant moderator. We further investigated whether age moderated the relationship between perceived usefulness and behavioural intention, and we found that age was a significant moderator (See Fig 1). More specifically, it was found that when people, irrespective of age, considered digital health technology as useful (at a high and medium level) they were more likely to have higher intention to use digital health technology. We also found that when digital health technology was perceived as less useful, individuals were more likely to report lower intentions to use digital health technology. This effect was amplified by age whereby younger participants reported higher intentions and older participants lower intentions to use digital health technology.

**Table 6:** Perceived Ease of Use Predicting Behavioural Intention with Age as a Moderator.

	Coeff	t	p	LLCI	ULCI	F	p
Model 1						87.99	< .001
Perceived Ease of Use	.50	3.09*	.002	.18	.82		
Age	-.05	.86	.387	-.15	.06		
Perceived Ease of Use*Age	.0008	.32	.752	-.001	.006		
Model 2						153.51	< .001
Perceived Usefulness	.32	2.26	.024	.06	.61		
Age	-.13	2.64	.009	-.23	-.33		
Perceived Usefulness * Age	.005	2.20	.029	.001	.009		

### Digital health technology used

About 20.7% (*n* = 40) of participants reported that they do not use any digital health technology. Of these, some reported they *‘hate it’* and while others lacked awareness of any. About 11.4% (*n* = 26) said that they use ‘Patient Knows Best’, although a few described their dislike for it. About 5.2% (*n* = 22) said that they use ‘Patient View’, with one saying how they preferred it to ‘Patient Knows Best’. About 10.9% (*n* = *25*) said that they either use a smartwatch (e.g. apple watch or Fitbit) or a phone app (e.g. MyFitnessPal) to monitor general health on their phones. A large proportion, 29.7% (*n* = 68), did not name a digital health technology but stated that they were accessing their own medical records through the internet, phone speaking to GP. Of this proportion of participants, one individual reported they would welcome a digital health technology that monitors Creatine, and another stated a preference for accessing healthcare through “people” i.e. talking directly to health care professionals. A total of 3.5% (*n* = 8) use MyRenalCare.

## Discussion

This study found that people with CKD were more likely to perceive digital health technology as easy to use if they also believed that there are organisational and technical resources to support the use of digital health technology and if they reported less anxiety and a high sense of enjoyment towards the use of digital health technologies. We also found that people with CKD considered digital health technology as useful if they also perceived it to be easy to use and relevant to their care. Furthermore, we found that people with CKD reported greater intention to use digital health technology if they perceived it to be easy to use and useful to their overall care. Finally, when digital health technology is not perceived particularly useful, people with CKD reported lower intentions to use digital health technology and this effect was amplified by age, whereby younger participants reported higher intentions and older participants reported lower intentions to use digital health technology.

Based on a large cohort UK study, our sample was not representative of the UK population that have a diagnosis of CKD, although there were some similarities [33]; our sample was younger 61 years of age versus 74 years of age. The gender distribution was similar with predominately more females and majority of the participants were white. Our study differed in the income and education status between the two samples. In previous European studies, younger people (16–24 years of age) predominated in the group with higher basic digital skills and 64-75-year-olds predominated in the group with the lowest basic digital skills [34,35]. This is consisted in UK studies too that have found older people had lower positive attitudes towards the digital health technologies [9]. CKD is more prevalent in older age groups, which presents a particular challenge in this cohort [36] but can further widen the gap in inequalities to access to healthcare [9]. Accordingly, people with CKD may require additional input to facilitate adoption of digital health technology. This could be provided by their clinical care teams, their families or via charities involved with CKD. Such help may comprise (a) explaining what digital health technology is; (b) how it can be utilised in the care of CKD (c) what the benefits are of using digital health technology and (d) helping them navigate the technology [36].

Although MyRenalCare is the system used in Wessex, in this survey we did not specify any particular digital health technology product, thereby gaining insights into digital healthcare technology in general. We found that an individual’s perceptions the effort involved in the use of digital health technology is a key determinant of their intention to use it. This is consistent with previous research showing that people with HIV are more likely to embrace mhealth if it is easy to use [37]. We found that technology is considered easy to use if individuals perceived it to have good organisational and technical support and if they reported less anxiety and greater enjoyment when using it. Accordingly, when developing future digital health technologies, it is important that users (people with CKD and healthcare professionals) are confident that a robust technology infrastructure is in place to assist when issues arise. This is supported by a previous evaluation of apps, where the lowest rating was recorded for apps prone to technical difficulties [38]. The best rated apps were those co-developed by expert organisations (e.g. National Kidney Foundation, US) suggesting that specialist insight into the needs of patients with specific medical problems is important when developing digital systems. Generic systems crossing several disease areas may therefore not be the optimal solution.

In a previous systematic review, perceived ease of use and perceived usefulness were the most common factors that influenced use of the mHealth system [39]. The findings of our study are consistent with this. As digital health technologies are a new development, the usual factors determining perception of usefulness cannot apply; i.e. widespread use (subjective norm) stats attached to people who use it (image) or effectiveness of the technology (output quality). It is assumed that with the uptake of digital technology, the relevance of these factors will increase in future.

### Implications for future research and practise

Hitherto, the acceptability of specific digital health technology systems has not been unexplored in people with CKD. As more digital health technologies, in the form of websites [40] start to populate the market it is important to explore the suitability and acceptability of each from the perspective of both patients and healthcare providers. As well as explore the practical implication of what it means to use those digital health technologies in the care of people. Elements such as training to learn how to use it, time to use it and other factors will need to be further explored. However, a key to this is evaluating the importance of usefulness and relevance of digital systems to specific patient groups.

In a more practical level, the results of the current study indicate that if organisations like the NHS or healthcare professionals working in hospitals start adopting the use of digital health technologies, they will need to make sure that there is enough robust technical support in place to address issues that users encounter. This is particularly important for older users that a number of conditions affect e.g. CKD, dementia, but healthcare professionals too that will be using the digital health technology. It is essentially important to provide support to the health professionals and the clinical teams, given that the support for the older people will need to be facilitated by the clinical care teams, families, and relevant charities that focus on the education about the technology. Additionally, digital health technologies will have to go through a series of tests to make sure that they have user-friendly interfaces to minimise anxiety with its use. While also have engaging interactive features to make the users motivation in using it. Using a co-creation approach is important to ensure that they meet the specific needs of the people with CKD.

### Strengths and limitations

A limitation of the current study is the high number of people who did not access the link to take part in the survey. Although the number who did not complete the survey was high (39%) this was in the range of previous studies exploring app-based intervention in chronic disease (43%; [41]. It is possible that using a different means of collecting information (e.g. by post) might improve data collection, particularly from people with poor computer skills (who may have been disproportionately excluded from the data). The list of the 12,000 people who received the invitation to participate all had an email address, which again may have excluded people who are not skilled in computer use. Another limitation of the study is that it is exploring digital health technology in general from a group of people who may have used only a specific system. Their impressions may have been coloured by their experience. To mitigate this, the questions used were intentionally generic in nature and avoided any mention of MyRenalCare.

## Conclusion

Previous studies have shown that whist the use of digital health technology is increasing, its use in older age groups remains low [42]. There is therefore potential for an increase in health inequalities. [43]. Given that CKD predominantly, also affects older adults, it is essential to provide targeted support to this cohort to facilitate the adoption of digital health technologies. This support can come from clinical care teams, families, and relevant charities, focusing on education about the technology, its benefits, and practical guidance on its use. It is therefore important to explore how systems can be created which reduce this potential. In the current study we did not find that age itself determined the likelihood that digital health technology would be adopted; instead, we found that older people had less intention to use digital health technology if they did not see it as useful for their care. This indicates that if the perceived usefulness of digital health technology is communicated appropriately, the intention of people with CKD to use digital health technology should improve. This highlights the importance of co-developing apps and digital health technology with the intended end users; to do so allows key barriers to be identified and addressed.

Future research should continue to explore the acceptability and factors influencing the use of specific digital health technology and how they can be integrated to the health care system from the perspectives of everyone involved (e.g. users, carers, healthcare professionals, clinical teams, charities). By addressing all of these factors and stakeholders the development and implementation of digital health technologies can be optimised to better serve people with CKD ultimately improving their health outcomes and quality of life.
